# Controlled Low‐Oxygen Supply Enables Magnetosome Size Tuning by Uncoupling Magnetite Nucleation and Crystal Growth in *Magnetospirillum gryphiswaldense*

**DOI:** 10.1111/1751-7915.70349

**Published:** 2026-04-14

**Authors:** Sophia Tessaro, Markus Schüritz, Valérie Jérôme, Ruth Freitag, René Uebe

**Affiliations:** ^1^ Department of Microbiology, Faculty of Biology, Chemistry and Geosciences University of Bayreuth Bayreuth Germany; ^2^ Department of Process Biotechnology, Faculty of Engineering Sciences University of Bayreuth Bayreuth Germany

## Abstract

The magnetotactic bacterium *Magnetospirillum gryphiswaldense* MSR‐1 synthesizes membrane‐enclosed magnetite (Fe_3_O_4_) nanocrystals, known as magnetosomes. Owing to their uniform size, purity and superior magnetic properties, magnetosomes represent highly attractive nanomaterials for biotechnological and biomedical applications. However, their bioproduction is limited by demanding cultivation requirements, largely because magnetite biomineralization is highly sensitive to environmental parameters, particularly oxygen. While elevated oxygen concentrations are known to inhibit magnetosome formation, quantitative analyses under defined low‐oxygen conditions are scarce. Here, we cultivated MSR‐1 in bioreactors under precisely controlled dissolved oxygen (DO) levels and quantified growth behaviour, substrate uptake and magnetosome characteristics. Cells harvested during late exponential growth revealed that magnetite crystal numbers per cell were similar across a wide DO range (0%–5%), whereas crystal sizes decreased with increasing oxygen levels. The data further indicate that oxygen inhibits biomineralization primarily through direct oxidative interference rather than indirect metabolic effects. These findings provide a mechanistic basis for optimizing oxygen control strategies in MTB cultivation and demonstrate that fine‐tuning DO levels enables targeted modulation of magnetosome size and properties. This advances both the bioprocess development of high‐yield magnetosome production and the application of tailored magnetic nanoparticles in biotechnology and medicine.

## Graphical abstract

Precisely controlled low‐oxygen conditions uncouple magnetite nucleation from crystal growth in *Magnetospirillum gryphiswaldense* , enabling targeted tuning of magnetosome size and magnetic properties. This provides a mechanistic basis for rational bioprocess optimization of magnetosome production.

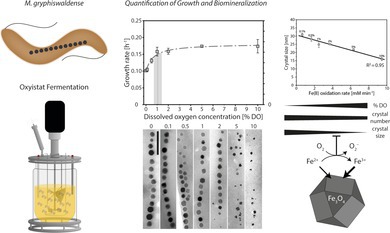

## 1 Introduction

The magnetotactic alphaproteobacterium *Magnetospirillum gryphiswaldense* MSR‐1 biosynthesizes magnetosomes, membrane‐enclosed nanocrystals of magnetite (Fe_3_O_4_) that enable geomagnetic navigation towards preferred microoxic niches. Biosynthesis of these specialized organelles is mediated by ~30 magnetosome‐associated proteins (MAPs), which coordinate the stepwise biogenesis pathway and precisely regulate the physicochemical conditions within the magnetosome lumen (Uebe and Schüler 2016). This control enables the biomineralization of chemically pure, stoichiometric magnetite crystals with narrow size and shape distributions, which confer magnetosomes with strong magnetic properties (Fischer et al. 2011). In addition, the protein‐rich magnetosome membrane enhances colloidal stability and can serve as a versatile platform for selective functionalization with enzymes, fluorophores or antibodies (Vargas et al. 2018). Moreover, genetic engineering allows modulation of the magnetite core size to fine‐tune the magnetic behaviour of magnetosomes from stable single magnetic domain (i.e., *Ø* > 30 nm) to superparamagnetic (i.e., *Ø* < 30 nm) particles (Mickoleit and Schüler 2019). Owing to these combined properties, magnetosomes are superior to abiogenic nanoparticles (Schüler et al. 2025), whose synthesis often relies on expensive organometallic precursors, harsh reaction conditions or toxic high‐boiling solvents (Lenders et al. 2017). Magnetotactic bacteria such as MSR‐1 therefore represent an eco‐friendly, low‐temperature alternative for the production of magnetite nanoparticles with high biotechnological and biomedical relevance.

Isolated magnetosomes have been successfully applied in anti‐cancer therapies via magnetic hyperthermia (Gandia et al. 2019), photothermia (Plan Sangnier et al. 2018) or magnetically guided drug delivery (Geng et al. 2019). They also outperform synthetic nanoparticles as contrast agents in MRI (magnetic resonance imaging) (Mériaux et al. 2015; Boucher et al. 2017) and as tracers in MPI (magnetic particle imaging) (Kraupner et al. 2017). Importantly, these imaging modalities require distinct magnetic particle sizes. While small single‐core superparamagnetic crystals (25–30 nm), which provide a strong dynamic magnetization response without remanence, are optimal for MPI (Heinke et al. 2017; Kraupner et al. 2017; Mickoleit et al. 2023), larger ferrimagnetic particles (> 40 nm) are advantageous for MRI due to their higher magnetic moment and associated transverse relaxation effects (Taukulis et al. 2015). Reproducible bioproduction of magnetosomes with defined core sizes is therefore essential for clinical translation.

However, routine flask cultivation of MSR‐1 often results in substantial batch‐to‐batch variability in magnetosome size (Mickoleit et al. 2023), likely due to uncontrolled supply of the key process parameter oxygen. In MSR‐1 and related species, oxygen concentrations above ~20 mbar (corresponding to DO concentrations > 10% air saturation), for example, inhibit biomineralization (Heyen and Schüler 2003), whereas low‐oxygen levels (~0.1%–5% DO) promote magnetosome formation (Blakemore et al. 1985; Schüler and Baeuerlein 1998; Yang et al. 2001; Heyen and Schüler 2003). The strong influence of oxygen on crystal formation is further supported by observations in the closely related species *Paramagnetospirillum magneticum* AMB‐1, where varying shaking frequencies during flask cultivation and thus oxygen supply revealed that magnetite crystal size decreases with increasing oxygen availability (Li and Pan 2012).

Although early studies suggested that oxygen is essential for magnetite biomineralization (Blakemore et al. 1985), isotopic labelling later demonstrated that the oxygen atoms in magnetite originate from water rather than molecular O_2_ (Mandernack et al. 1999). Together with the observation that MSR‐1 requires several hours to initiate magnetosome formation after a shift from inhibitory to permissive oxygen levels, it was therefore proposed that oxygen acts as a regulatory signal, potentially by inducing protein synthesis (Heyen and Schüler 2003). However, consistent with the presence of empty magnetosome vesicles under aerobic conditions (Raschdorf et al. 2016), transcriptomic analyses show that MAP gene expression is oxygen‐independent (Wang et al. 2016, 2017; Riese et al. 2022). These findings thus indicate that oxygen interferes directly with biomineralization, possibly by oxidizing ferrous iron required for magnetite formation or by inactivating redox‐sensitive proteins or cofactors essential for biomineralization (Schüler et al. 2025). In line with this view, strictly anoxic cultivation of the facultative anaerobe MSR‐1 yields larger and more well‐defined magnetite crystals (Riese et al. 2020).

Beyond relieving oxygen inhibition, anoxic or low microoxic conditions may also indirectly promote biomineralization by upregulating the respiratory denitrification pathway (Riese et al. 2022). This pathway converts nitrate to dinitrogen gas and has repeatedly been associated with enhanced magnetite formation. For instance, *Paramagnetospirillum magnetotacticum* MS‐1 produced more magnetosomes under microoxic conditions when nitrate rather than ammonium was supplied as the nitrogen source (Blakemore et al. 1985). Conversely, depletion of molybdenum, an essential cofactor of the periplasmic nitrate reductase Nap (i.e., the first enzyme of denitrification), reduced the cellular iron content of *P. magnetotacticum* MS‐1 by ~60% (Taoka et al. 2003). Similarly, genetic disruption or deregulation of key denitrification enzymes also severely impaired magnetite biomineralization in MSR‐1 (Li et al. 2012, 2013, 2014; Silva et al. 2020). Although the molecular mechanisms remained unclear, it has been proposed that denitrification contributes to magnetite biomineralization by establishing suitable intracellular redox conditions via the nitrate reductase Nap (Li et al. 2012). Alternatively, the nitrite reductase NirS may modulate the ferrous‐to‐ferric iron ratio through its Fe(II)‐NO_2_^−^ oxidoreductase activity (Yamazaki et al. 1995).

In summary, oxygen availability has strong direct and indirect effects on magnetite biomineralization, but quantitative insights under controlled microoxic cultivation conditions remain limited to only a few conditions (Riese et al. 2020). To address this gap, we performed systematic bioreactor cultivations of MSR‐1 under defined low DO concentrations and quantified growth, substrate uptake and magnetosome formation. Our data are consistent with robust magnetite nucleation between 0% and 5% DO, whereas crystal growth appears to be strongly inhibited even at very low‐oxygen levels. These findings identify oxygen as the key process parameter that uncouples magnetite nucleation from crystal growth and therefore determines magnetosome size. This mechanistic insight provides a foundation for future bioprocess optimization and for the tailored production of magnetosomes with defined magnetic properties.

## 2 Experimental Procedures

### 2.1 Bacterial Strain and Growth Conditions

In this study, wildtype *M. gryphiswaldense* MSR‐1 (DSM 6361) was used throughout all experiments. For routine cultivations, MSR‐1 was incubated for 24 h at 28°C with moderate agitation (120 rpm) in 15‐mL Falcon tubes containing 10 mL of modified flask standard medium (FSM). The medium consisted of 10 mM 2‐(4‐[2‐hydroxyethyl] piperazin‐1‐yl) ethanesulfonic acid (HEPES), 15 mM potassium lactate, 4 mM sodium nitrate, 0.74 mM potassium dihydrogen phosphate, 0.6 mM magnesium sulphate heptahydrate, 150 μM iron citrate, 3 g L^−1^ soy peptone and 0.1 g L^−1^ yeast extract (pH 7.0) (Heyen and Schüler 2003).

For fermentation experiments, MSR‐1 was passaged in FSM at least four times before preculture inoculation. To suppress magnetosome formation, the final two passages were carried out under oxic conditions. Cells were first grown in 100 mL FSM in 1‐L baffled shake flasks at 28°C and 120 rpm for 16 h. Subsequently, 30 mL of the exponentially growing culture were transferred to 300 mL fresh FSM in a 2‐L baffled flask and incubated under the same conditions for another 16 h. The resulting essentially non‐magnetic precultures were then used to inoculate the 3‐L bioreactors. This seed train, that is, the stepwise scale‐up through successive precultures, ensured that the inoculum biomass was at a consistent cell density and in a comparable metabolic state, thereby improving reproducibility.

### 2.2 Bioreactor Setup

In initial experiments, a BioLector I microbioreactor (m2p‐labs, Baesweiler, Germany) able to monitor but not regulate pH, DO and biomass online was used to screen growth of MSR‐1 in FSM under high‐throughput conditions. To ensure efficient oxygen transfer, cultures were shaken at 800 rpm in flower‐shaped 24‐well plates containing 1 mL medium. All cultures were inoculated to an initial optical density (OD_565 nm_) of 0.01 from a preculture of MSR‐1 that had been subcultivated at least four times. The plates were then incubated at 28°C for 2 days under atmospheric oxygen conditions.

All subsequent experiments were conducted in 3‐L, double‐jacketed BioFlo 320 bioreactors (Eppendorf Bioprocess, Jülich, Germany) equipped with four baffles, a Rushton impeller positioned 1.3 cm above the shaft end and a pitched‐blade impeller located 4 cm above it. The control units of the bioreactor system were fitted with thermal mass flow controllers for gas inlet. To monitor the pH during cultivation, an InPro3253i pH sensor (Mettler‐Toledo, Columbus, USA) was used. The pH setpoint was maintained at 6.95 to counteract medium alkalinization caused by the denitrifying metabolism of MSR‐1. A dead‐band of 0.1 pH units was applied, and pH control was achieved by automated addition of 1 M sulphuric acid (except for anoxic experiments where nitric acid was used) and 1 M sodium hydroxide. Online measurement of DO (reported as % air saturation) was carried out by an InPro6850i DO sensor (Mettler‐Toledo, Columbus, USA) with a lower limit of six parts per billion at an accuracy of ±1%. After sterilization and cooling of the fermenter to the corresponding process temperature (28°C, except for temperature‐dependent experiments), the medium was sparged with nitrogen until measured current values were stable (0% DO). Afterwards, the medium was sparged with sterile air to achieve saturation (100% DO) for O_2_ probe calibration. DO was regulated using a cascade in which the PI controller output sequentially increased impeller speed (100–300 rpm) and aeration rate (0–10 standard litres per minute) (Riese et al. 2020). All parameters (temperature, DO, gas flow, agitation rate, pH, added acid and base volume) were monitored online and recorded by the Biocommand software (Eppendorf Bioprocess, Jülich, Germany).

For bioreactor experiments, we used 2.5 L FSM containing either 50 or 150 μM (only during anoxic experiments) iron citrate which caused no difference in magnetosome size distribution patterns (Figure S1A). To compensate for the increased nitrate demand under anoxic or low microoxic conditions, nitrate concentrations were raised to 8 mM (0.5% DO), 10 mM (0.1% DO) or 12 mM (0% DO). At 0% DO, nitric acid was additionally used as the pH control agent. Microfermentation assays confirmed that these elevated nitrate levels did not alter growth compared to standard medium (B).

Fermentations were started by inoculation of 330 mL exponentially growing, essentially non‐magnetic precultures. Growth of the cultures was then measured turbidimetrically with an Ultrospec 2100 pro spectrophotometer (GE Healthcare, Buckinghamshire, UK) at a wavelength of 565 nm every 2–4 h (Dziuba et al. 2020). The growth rate *μ* was then calculated from two consecutive data points (equation 1):

$$\mu =\frac{\ln\frac{{\mathrm{OD}}_{2}}{{\mathrm{OD}}_{1}}}{t_{2}-t_{1}}$$

After each OD measurement, culture samples were diluted to an OD of 0.1 to measure the cellular magnetic response C_mag_, which provides a semiquantitative measure of magnetosome formation based on differential light scattering of cells in a magnetic field (Schüler et al. 1995). Therefore, a magnet was positioned parallel (OD_max_) and perpendicular (OD_min_) to the light beam of the photometer, and C_mag_ was calculated according to equation 2:

$$C_{\mathrm{mag}}=\frac{{\mathrm{OD}}_{\max}}{{\mathrm{OD}}_{\min}}-1$$

### 2.3 Substrate Analytics

For substrate analytics, 15 mL culture samples were harvested at regular intervals throughout the fermentation experiments. Subsequently, the samples were centrifuged for 15 min at 8000 × g to pellet the cells, and the cell‐free supernatant was transferred to a new tube and stored at −20°C until further use.

Frozen, cell‐free culture supernatants were thawed and analysed for iron, lactate and nitrate concentrations to determine substrate consumption rates. Iron concentrations were measured using a contrAA300 high‐resolution atomic absorption spectrometer (Analytik Jena, Jena, Germany) as described (Rosenfeldt et al. 2021). Nitrate concentrations were measured with a Spectroquant Nitrate Kit (Merck KGaA, Darmstadt, Germany) or a Szechrome NAS Quantification Kit (Polysciences Inc., Warrington, USA) according to the manufacturer's instructions. Additionally, the concentration of the denitrification intermediate nitrite was measured using a Griess reagent kit (Sigma‐Aldrich Chemie GmbH, Steinheim, Germany) as described recently (Woller et al. 2026). Lactate was measured by an enzymatic amperometric approach using the Biosen S‐Line Lab+ analyser according to the manufacturer (EKF‐Diagnostic, Barleben, Germany).

### 2.4 Transmission Electron Microscopy (TEM)

For TEM analysis, cells were sampled at an OD of ~1 (13–30 h post‐inoculation, depending on conditions) and fixed with 0.3% (w/v) formaldehyde. Sampling at the same growth phase ensured that magnetosome formation could be compared across cultures with differing growth rates and metabolic activities. Fixed cells were then deposited on carbon‐coated copper grids (Science Services, Munich, Germany), washed twice with ddH_2_O and air‐dried (Zwiener et al. 2021). Images were taken with a JEM 1400+ (Jeol GmbH, Freising, Germany) operating at 80 kV voltage (Dziuba et al. 2023). Particle sizes were measured manually using the ImageJ software (V2.1.0/1.53c) (Schindelin et al. 2012).

### 2.5 Statistical Analyses

Data were subjected to statistical analyses with the Past software (version 4.03) (Hammer et al. 2001). A *p*‐value of less than 0.05 was considered statistically significant. TEM‐based data for quantification of magnetosome size and number per cell were pooled across replicates prior to statistical analyses. Summary statistics of individual replicates are provided in Tables S1–S4. Further information about statistical details and methods is indicated in the figure captions, text or methods. If not stated otherwise, values are given as mean ± standard deviation of the indicated sample size. Plots and fit functions were generated with the Fit‐o‐Mat 0.944 software (Möglich 2018).

## 3 Results

### 3.1 Small‐Scale Batch Cultivation Setups Fail to Maintain Stable DO and pH

To assess the influence of oxygen on growth and magnetosome formation in MSR‐1, we initially aimed to use a microbioreactor system capable of monitoring biomass, DO and pH in real time. Batch cultivation experiments at the 1‐mL scale with this system, however, revealed that even at very low cell densities (starting OD = 0.01), neither the medium pH nor the DO could be maintained at a constant level. Under aerobic growth conditions, the pH of the HEPES‐buffered flask standard medium (FSM, pH 7) began to increase at the onset of the exponential growth phase (after ~10 h) and reached a final pH of 8.9 after 43 h (Figure S2). Similarly, despite continuous shaking at 800 rpm within flower‐shaped 24‐well plates to facilitate oxygen dispersion and transfer, the DO level also started to decrease after 10 h and was fully depleted after 27 h. These changes resulted in a multiphasic growth curve in which two ~10 h exponential growth phases were interrupted by two short stationary phases 18–23 h after inoculation (Figure S2). Together, these findings highlight the need for precise environmental control when assessing oxygen effects on growth and magnetosome formation, as pH has likewise been shown to influence growth and magnetite biomineralization in MSR‐1 (Moisescu et al. 2011). Therefore, all subsequent experiments were conducted in 3‐L bioreactors.

To this end, we used a previously established fermentation setup that enabled highly consistent growth experiments by precisely controlling dissolved oxygen, pH and temperature throughout the process (Table S5) (Riese et al. 2020). For a quantitative assessment of biomineralization under the tested conditions, we additionally developed an oxic seed train protocol to obtain magnetite‐free precultures. This allows analysis of magnetosome formation during the cultivation in bioreactors with a minimized influence of crystal‐containing cells from the inoculum. Therefore, magnetic precultures were grown twice in baffled shake flasks at 120 rpm until they reached an OD of ~0.5 (after ~16 h) and a cellular magnetic response (C_mag_) of ~0. TEM analyses confirmed that cells contained approximately one magnetite crystal at the end of the seed train (mean 0.6 ± 0.9 crystals per cell, *N* = 142 cells; Figure S3).

All bioreactor experiments were then conducted in triplicates to ensure robustness. In addition, a reference culture grown under standard conditions (28°C, pH 7, 1% DO) used previously (Riese et al. 2020) was included to test for potential batch‐to‐batch variation.

### 3.2 Magnetite Crystal Growth Is Impaired by Low‐Oxygen Levels

Earlier studies showed that magnetite biomineralization is completely inhibited at DO levels above 10% (Heyen and Schüler 2003). We therefore performed batch cultivations at DO concentrations between 0% and 10%, while maintaining pH 7 and 28°C.

**FIGURE 1:**
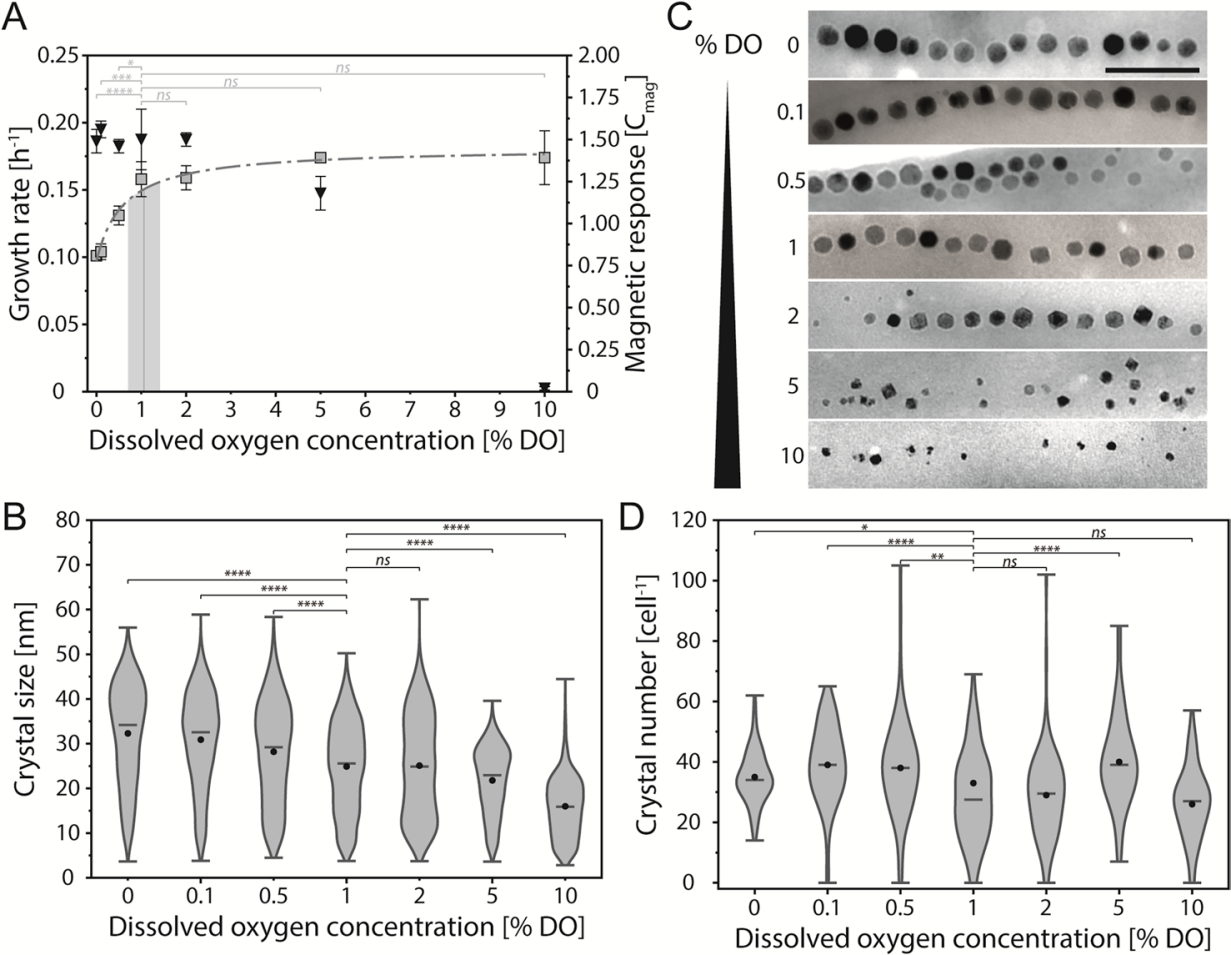
Effect of oxygen on MSR‐1 growth and magnetosome formation. (A) Growth rate (squares) and cellular magnetic response (triangles, C_mag_) in dependence of dissolved oxygen concentrations. The growth rate follows a modified Monod kinetic model (dash dotted line, *R* ^2^ = 0.973). The light grey bar represents the preferred localization of MSR‐1 cells within aerotactic bands in oxygen gradients, with the thin line denoting the mean position (data taken from Bennet et al. 2014). (B) Violin plot displaying the magnetite crystal size distribution depending on the dissolved oxygen concentration. Crystal sizes, given in nm, were measured from TEM micrographs. The min, max and median values are indicated by bars, mean values by black dots. The number of analysed cells is 90 (0% DO), 78 (0.1% DO), 81 (0.5% DO), 98 (1% DO), 86 (2% DO), 75 (5% DO) and 72 (10% DO), respectively. (C) Representative TEM micrographs of magnetite crystals from cells cultivated at different dissolved oxygen concentrations. Scale bar, 200 nm. (D) Violin plot showing the distribution of magnetite crystal numbers per cell depending on dissolved oxygen concentration. Crystals per cell were determined from TEM micrographs. Data displayed similar (B). The number of analysed cells is 3143 (0% DO), 3078 (0.1% DO), 3087 (0.5% DO), 3681 (1% DO), 2541 (2% DO), 3021 (5% DO) and 1902 (10% DO), respectively. Statistical significance in A was calculated by Tukey's *Q*‐test, significance in B and D was estimated using the Dunn's post hoc test with the Bonferroni correction. Asterisks indicate the level of significance: *, *p‐*value ≤ 0.05; **, *p*‐value ≤ 0.01; ***, *p*‐value < 0.001; ****, *p*‐value < 0.0001; ns, not significant.

In our experiments, anoxic conditions resulted in lowest MSR‐1 growth rates (*μ* = 0.10 ± 0.002 h^−1^, ~60% of reference) (Figure 1A). With increasing oxygen levels, growth rates were significantly enhanced, reaching near‐maximum levels at 1% DO (*μ* = 0.16 ± 0.01 h^−1^) and peaking at 5% DO (*μ* = 0.17 ± 0.002 h^−1^). Accordingly, the growth rate followed a modified Monod model with an apparent half‐saturation constant (*K*_s_) of 0.6% ± 0.2% DO, indicating that DO levels above 1% do not significantly improve growth (*p* > 0.55, Tukey's *Q*‐test, Figure 1A).

In contrast to growth, the cellular magnetic response was similar between 0% and 2% DO (C_mag_~1.5), decreased to 1.2 ± 0.1 at 5% DO and remained negligibly low (C_mag_ 0.02 ± 0.01) at 10% DO (Figure 1A). While these results indicate that oxygen starts to impair magnetite biomineralization above 2% DO, TEM analyses of cells sampled at the late exponential growth phase (OD ~1) revealed a clear influence of oxygen on magnetosome crystal size even at low DO levels. Under anoxic conditions, cells contained magnetite crystals with a mean diameter of 32.3 ± 11.1 nm. The size decreased to 30.9 ± 10.6 nm at 0.1% DO (*p* = 0.003 vs. anoxic, Dunn's post hoc test) and 28.2 ± 11.3 nm at 0.5% DO (*p* = 1 × 10^−42^ vs. anoxic, Dunn's post hoc test) (Figure 1B). Higher DO levels resulted in further reduction of crystal diameters with smallest particles formed at 10% DO (mean diameter 15.7 ± 6.6 nm). Additionally, particles formed at 5% DO occasionally and at 10% DO frequently exhibited shape‐defects (Figure 1C). Notably, crystal numbers per cell were only modestly affected by the presence of oxygen. Cells grown at 10% DO contained only ~25% fewer crystals compared with cells cultivated under anoxic conditions (Figure 1D). In summary, our data indicate that magnetite crystal growth, but not nucleation, is sensitive to low‐oxygen levels and is maximal under strictly anoxic conditions, which are suboptimal for cell growth.

### 3.3 Iron Uptake, Not Denitrification, Correlates With Magnetite Crystal Size

Next, to correlate the MSR‐1 denitrification activity with the applied oxygen levels and magnetite formation, we quantified the nitrate consumption of MSR‐1 and tested for transient accumulation of the intermediate nitrite. In addition, we analysed whether carbon source (lactate) and iron‐uptake rates also correlate with biomineralization. Notably, lactate uptake was lowest under anoxic conditions (1.2 ± 0.1 mM h^−1^ OD^−1^) and increased two‐fold to 2.4 ± 0.1 mM h^−1^ OD^−1^ at 1% DO. At higher DO levels, uptake increased only slightly, reaching a maximum of 2.8 ± 0.1 mM h^−1^ OD^−1^ at 10% DO (Table 1). Thus, the specific lactate uptake rate closely mirrored the dependence of the growth rate on DO.

**TABLE 1:** Specific substrate‐uptake rates of MSR‐1 during growth at different DO levels. Values are given as mean ± standard deviation calculated from three independent experiments. *ND*, not determined.

DO [%]	Lactate uptake rate [mM h^−1^ OD^−1^]	Nitrate uptake rate [mM h^−1^ OD^−1^]	Iron‐uptake rate [μM h^−1^ OD^−1^]
0	1.2 ± 0.1	2.1 ± 0.2	7.2 ± 0.2
0.1	1.4 ± 0.1	1.0 ± 0.2	*ND*
0.5	1.9 ± 0.1	0.5 ± 0.1	7.7 ± 0.8
1	2.4 ± 0.1	0.4 ± 0.1	6.5 ± 1.2
2	2.7 ± 0.2	0.3 ± 0.1	5.5 ± 0.2
5	2.6 ± 0.1	0.5 ± 0.1	5.6 ± 1.4
10	2.8 ± 0.1	0.4 ± 0.2	3.1 ± 0.4

In contrast, nitrate uptake was highest under anoxic conditions (2.1 ± 0.2 mM h^−1^ OD^−1^) and decreased strongly with rising DO, resulting in a ~4‐fold lower nitrate uptake at 0.5% DO (0.5 ± 0.1 mM h^−1^ OD^−1^) (Table 1). Beyond this DO level, nitrate uptake remained relatively constant (0.3–0.5 mM h^−1^ OD^−1^ between 0.5% and 10% DO). At all tested oxygen levels, nitrite accumulated transiently at concentrations of 7–187 μM per mM of nitrate consumed, indicating that denitrification occurs under all tested oxygen levels.

Iron uptake was maximal under anoxic (7.2 ± 0.2 μM h^−1^ OD^−1^) and low microoxic conditions (7.7 ± 0.8 μM h^−1^ OD^−1^ at 0.5% DO) and declined progressively with increasing oxygen, reaching a minimum at 10% DO (3.1 ± 0.4 μM h^−1^ OD^−1^) (Table 1). Accordingly, the specific iron‐uptake rate was strongly correlated with magnetite crystal size (the Pearson correlation coefficient *r* = 0.92, *p* = 0.01) (Figure S4A). In contrast, specific lactate and nitrate uptake rates showed no significant correlation with crystal size (Figure S4B), suggesting that denitrification influences magnetite biomineralization only indirectly, or that its beneficial effect is already saturated at low activities.

### 3.4 Reduced Growth Rates Do Not Significantly Affect Magnetite Biomineralization

Earlier studies proposed that slower growth promotes more controlled biomineralization (Faivre et al. 2008; Katzmann et al. 2013). The larger magnetite crystals observed under strictly anoxic conditions may therefore also result from reduced growth rates (Figure 1). Consistent with this idea, we observed a strong linear relationship (*R* ^2^ = 0.91) between the growth rate and mean magnetite crystal sizes within the ~20–35 nm range (Figure S5A). To test this relationship independently of oxygen availability, we performed a second set of experiments in which growth rates were modulated by varying the incubation temperature.

**FIGURE 2:**
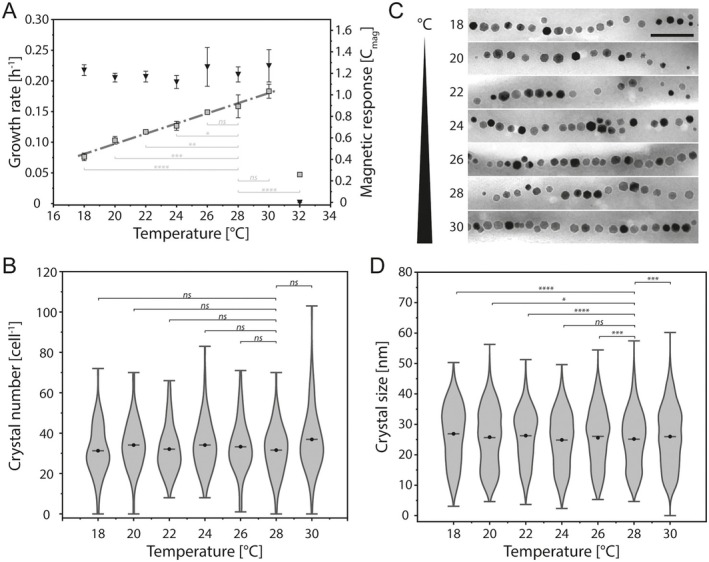
Effect of cultivation temperature on MSR‐1 growth and magnetosome formation. (A) Growth rate (squares) and cellular magnetic response (triangles, C_mag_) depending on the cultivation temperature. Between 18 and 30°C growth rates increase linearly (dash dotted line, linear regression with *R* ^2^ = 0.985). (B) Violin plot showing the distribution of magnetite crystal numbers per cell depending on the incubation temperature. Crystals per cell were determined from TEM micrographs. The number of analysed cells is 97 (18°C), 119 (20°C), 167 (22°C), 103 (24°C), 100 (26°C), 262 (28°C) and 88 (30°C), respectively. (C) Representative TEM micrographs of magnetite crystals from cells cultivated at different temperatures. Scale bar of 200 nm applies to all images. (D) Violin plot displaying the magnetite crystal size distribution depending on the incubation temperature. Crystal sizes, given in nm, were measured from TEM micrographs. The number of analysed cells 1967 (18°C), 4085 (20°C), 5358 (22°C), 3605 (24°C), 2342 (26°C), 8350 (28°C) and 3261 (30°C), respectively. Data in (B) and (D) is displayed similar to Figure 1B. Statistical significance in (A), (B) and (D) was calculated and depicted similar to Figure 1A,B,D, respectively.

To this end, we conducted cultivations at temperatures ranging from 18°C to 32°C, with increments of 2°C while otherwise maintaining standard conditions (1% DO, pH 7). Under these conditions, the growth rate increased linearly from 0.08 ± 0.01 h^−1^ at 18°C to 0.18 ± 0.01 h^−1^ at 30°C (Figure 2A). When the temperature was increased to 32°C, however, the growth rate decreased by more than 70% (*μ* = 0.05 ± 0.01 h^−1^) compared to 30°C, indicating a very narrow temperature range for optimal growth.

Between 18°C and 30°C, cultures exhibited highly similar cellular magnetic responses (C_mag_~1.2), and magnetite crystal sizes and numbers per cell showed only minor differences (Figure 2). Although some of these differences were statistically significant, no clear trend towards an optimal temperature for improved biomineralization was observed (Figure 2B,D, Figure S5B). In contrast, at 32°C neither a magnetic response nor magnetite crystals were detected. Thus, apart from the negative effect of elevated temperatures on growth and biomineralization, our induction setup revealed no impact of the incubation temperature, and thereby growth rate, on magnetite formation between 18°C and 30°C.

## 4 Discussion

Magnetite biomineralization by magnetospirilla has been extensively studied at the molecular level, whereas environmental parameters that influence the process have been tested only rarely so far. In this study, we systematically and quantitatively analysed how oxygen availability and incubation temperature affect biomineralization and cell growth in MSR‐1.

Consistent with previous reports (Heyen and Schüler 2003; Riese et al. 2020), magnetosome formation in MSR‐1 was optimal under oxygen‐limited conditions, with the largest magnetite crystals obtained under strictly anoxic conditions. Notably, even low‐oxygen levels led to a reduction of magnetite crystal size, while the number of crystals per cell was only minimally affected (Figure 1). Similar to observations in *P. magneticum* AMB‐1 (Popa et al. 2009), our data are consistent with magnetite nucleation being largely insensitive to oxygen across a broad concentration range, whereas subsequent crystal growth, as reflected by crystal size and shape, appears to be highly oxygen‐sensitive. This uncoupling of nucleation and crystal growth due to their differential oxygen sensitivity could be exploited to produce magnetosomes with precisely tuned sizes. For example, incubation at 2% DO yielded crystals of approximately 25 nm, which fall in the superparamagnetic size regime and are optimal for use as MPI tracers, but previously could only be achieved by genetic engineering (Mickoleit et al. 2023).

Because MAP‐encoding genes are constitutively expressed regardless of oxygen levels (Wang et al. 2016, 2017; Riese et al. 2022), crystal growth inhibition may result from direct interference of oxygen with the biomineralization pathway or indirectly from physiological changes associated with the anoxic or microoxic metabolism of MSR‐1. For example, components of the denitrification pathway have been proposed to mediate redox reactions that facilitate magnetite formation (Yamazaki et al. 1995; Li et al. 2012), thereby contributing to enhanced biomineralization under anoxic conditions.

To evaluate this possibility, we monitored nitrate consumption and transient nitrite accumulation. Consistent with earlier studies (Li et al. 2014; Riese et al. 2020), MSR‐1 performed denitrification in parallel with aerobic respiration across all tested oxygen concentrations. Compared to anoxic conditions, however, microoxic denitrification proceeds at substantially lower rates, as reflected by the strong decrease (~75%) in the specific nitrate uptake rate between 0% and 0.5% (from 2.1 ± 0.2 mM h^−1^ OD^−1^ at 0% DO to 0.5 ± 0.1 mM h^−1^ OD^−1^ at 0.5% DO). Notably, within this DO range the average magnetosome crystal size declined by only 13% (from 32.3 ± 11.1 nm to 28.2 ± 11.3 nm, *p* = 0.003). Thus, increased denitrification activities are unlikely to be a major determinant of enhanced biomineralization under anoxic conditions.

The crystallization defects observed at 5% and 10% DO, together with the increased production of non‐stoichiometric magnetite at elevated oxygen levels (Li and Pan 2012) and the impaired biomineralization of anoxic *P. magneticum* AMB‐1 cultures under high redox potentials (Olszewska‐Widdrat et al. 2019), suggest that oxygen may disrupt magnetite formation mainly by interfering with redox‐sensitive steps of the biomineralization pathway. Supporting this hypothesis, we found that the decrease in crystal size with rising oxygen levels was strongly negatively correlated with abiotic Fe(II) oxidation rates under low‐oxygen conditions (Pearson correlation coefficient r = −0.98, Figure S6) (Kanzaki and Murakami 2013). However, further analyses are required to distinguish between (i) abiotic oxidation of ferrous iron, which would reduce ferrous iron availability relative to ferric iron and thereby restrict growth of the mixed‐valence mineral magnetite, and (ii) oxygen‐dependent biochemical inactivation of redox‐sensitive magnetosome‐associated proteins, such as the haem‐containing magnetochrome‐domain proteins MamE, MamP, MamX and MamT (Siponen et al. 2012).

While anoxic conditions are optimal for magnetite biomineralization, the lower energy yield of the denitrification pathway (Strohm et al. 2007) resulted in significantly reduced growth of MSR‐1. Intermediate growth rates at 0.1% and 0.5% DO indicate that oxygen enhances energy yields through aerobic respiration, yet remains limiting in this concentration range. The strong decrease in nitrate uptake between 0.1% and 0.5% DO indicates a major shift in the relative contributions of anaerobic and aerobic pathways within this narrow interval. MSR‐1 approaches its maximal growth rate at 1% DO, indicating that aerobic respiration becomes saturated at this DO level, while denitrification resumes at a basal level. Interestingly, Bennet et al. (2014) observed that cells of MSR‐1 preferentially accumulate at oxygen concentrations between 1.5 and 3.6 μM O_2_ (corresponding to ~0.6%–1.4% DO, with a mean of 1.1% DO) to form so‐called aerotactic bands (data displayed in Figure 1A). Together with our findings, this suggests that the magneto‐aerotactic behaviour of MSR‐1 is tuned to detect the lowest oxygen concentration that still supports near‐maximal growth.

The analysis of growth at different temperatures revealed a directly proportional increase of the MSR‐1 growth rate up to 30°C, at which a maximum was reached. Further increase of the temperature to 32°C sharply reduced growth and abolished magnetite biomineralization. These findings indicate that MSR‐1 has a relatively narrow temperature range for optimal growth and a well‐defined upper limit for magnetosome formation under microoxic conditions. Complete inhibition of magnetite biomineralization at elevated temperatures (35°C) was likewise reported by (Moisescu et al. 2011), whereas (Katzmann et al. 2013) observed small polycrystalline particles under similar conditions. We attribute these discrepancies to differences in cultivation setups. Unlike our tightly controlled bioreactor experiments, both earlier studies used flask or Hungate cultures. As shown by our 1‐mL microbioreactor experiments, such uncontrolled batch setups do not maintain stable pH and oxygen levels. This likely reflects limited buffer capacity of the growth medium (FSM in all studies), which cannot fully compensate for metabolic alkalinization associated with denitrification, as well as high oxygen uptake rates that can rapidly deplete DO when oxygen transfer is insufficient (e.g., at low shaking intensity) or when gas–liquid exchange is restricted (e.g., in closed systems such as Hungate tubes). Consequently, unintended shifts in culture conditions may account for magnetite formation at 35°C under nominally aerobic conditions (Katzmann et al. 2013), despite the well‐established inhibitory effect of oxygen on biomineralization (Heyen and Schüler 2003).

Strikingly, between 18°C and 30°C, both the number of magnetosomes per cell and the average crystal diameter remained nearly constant, despite a 225% increase in growth rate (from 0.08 ± 0.01 h^−1^ at 18°C to 0.18 ± 0.01 h^−1^ at 30°C). Thus, although earlier studies proposed that slower growth and iron uptake might promote more controlled and improved biomineralization (Faivre et al. 2008; Katzmann et al. 2013), we found no such correlation under our induction conditions. Consequently, the increase in magnetite crystal size under anoxic conditions can be attributed to the absence of oxygen inhibition rather than to metabolic or growth rate‐dependent effects.

## 5 Conclusion

In summary, here we systematically quantified the effects of DO levels and incubation temperatures on growth and magnetite biomineralization in MSR‐1 using a strictly controlled bioreactor system. This approach provided deeper mechanistic insight into several aspects of magnetosome formation. For example, our results demonstrate that the enhanced biomineralization observed under anoxic conditions is not due to increased denitrification activity or reduced growth rates, but rather to the absence of inhibitory effects of oxygen on crystal growth. Combined with the strong correlation between oxygen‐dependent decreases in crystal size and abiotic Fe(II) oxidation rates, our findings also provide the first experimental evidence that oxygen directly inhibits magnetite crystal growth through ferrous iron oxidation. Furthermore, our results offer new insight into the ecophysiology of MSR‐1 by showing that its magneto‐aerotaxis targets microoxic conditions that maximize growth but are suboptimal for biomineralization. Implementing these findings in the rational design of cultivation strategies will enable reliable, high‐yield production of magnetosomes with defined core sizes and magnetic properties, thereby advancing their biotechnological and biomedical applications. Finally, our findings may also facilitate the modelling of bottom water oxygen concentrations in paleoclimatic reconstructions based on magnetofossils (Xue et al. 2025).

## Author Contributions

**Sophia Tessaro:** conceptualization, investigation, methodology, formal analysis, visualization, validation, data curation, writing – original draft. **Markus Schüritz:** methodology, investigation, data curation. **Valérie Jérôme:** methodology, investigation, data curation, writing – review and editing. **Ruth Freitag:** supervision, writing – review and editing. **René Uebe:** conceptualization, formal analysis, visualization, writing – original draft, review and editing, funding acquisition, project administration, supervision, resources.

## Funding

This work was supported by Bundesministerium für Bildung und Forschung, MagBioFab.

## Conflicts of Interest

The authors declare no conflicts of interest.

## Supporting information


**Figure S1:**. (A). Dependence of magnetite crystal size on the initial iron concentration under standard cultivation conditions. Statistical significance was calculated by a Mann–Whitney test. ns, not significant. (B) Growth of MSR‐1 in presence of different NaNO_3_ concentrations in 1 mL FSM.
**Figure S2:** Representative profiles of pH (purple triangles) and dissolved oxygen (blue circles) during aerobic growth of 1‐mL culture of MSR‐1 (orange squares) in a microbioreactor system at 28°C with constant vigorous shaking (800 rpm) and an initial pH of 7. Biomass was measured as the intensity of backscattered light (given in arbitrary units [AU]) from a light‐emitting diode. Coloured shading around the symbols indicates the standard deviation calculated from three independent experiments.
**Figure S3:** Representative MSR‐1 cell from the end of the second aerobic cultivation in the seed train.
**Figure S4:** Relationship between magnetosome crystal size and substrate‐uptake rates of MSR‐1 under varying dissolved oxygen (DO) levels. (A) Shows a positive correlation between iron‐uptake rate and magnetite crystal size. (B) No evident correlation between nitrate or lactate uptake rates and magnetite crystal size. Values are given as the mean ± standard deviation calculated from three independent experiments with the same DO level.
**Figure S5:** Correlation between magnetosome crystal size and growth rate of MSR‐1 during cultivation under different DO levels (A) and at different temperatures (B). Solid lines indicate linear regressions of data points with filled symbols (*R* ^2^ indicated in the upper left corner) whereas dashed lines represent no change in magnetite crystal size (25.8 nm) as a function of growth rate.
**Figure S6:** Correlation between magnetosome crystal size and abiotic Fe(II) oxidation rates under different DO levels. Oxidation rates were calculated for an initial iron concentration of 30 mM at pH 7.5 according to reference (Kanzaki and Murakami 2013).
**Table S1:** Summary statistics for TEM‐based magnetosome crystal size quantification for each biological replicate under the DO‐dependent cultivation conditions. *, approximate oxygen concentration at the selected DO setpoint.
**Table S2:** Summary statistics for TEM‐based magnetosome number per cell quantification for each biological replicate under the DO‐dependent cultivation conditions. *, approximate oxygen concentration at the selected DO setpoint.
**Table S3:** Summary statistics for TEM‐based magnetosome crystal size quantification for each biological replicate under the temperature‐dependent cultivation conditions.
**Table S4:** Summary statistics for TEM‐based magnetosome number per cell quantification for each biological replicate under the temperature‐dependent cultivation conditions.
**Table S5:** Summary statistics of process parameters during the active growth phase for each biological replicate (DO‐ and temperature‐setpoint experiments). Values are reported as time‐weighted mean ± time‐weighted mean absolute deviation.

## Data Availability

The data that supports the findings of this study are available in the supporting information of this article.
