# Supplementary material for: Controlled Low‐Oxygen Supply Enables Magnetosome Size Tuning by Uncoupling Magnetite Nucleation and Crystal Growth in Magnetospirillum gryphiswaldense
Source: Microb Biotechnol. 2026 Apr 14;19(4):e70349. doi: 10.1111/1751-7915.70349 (PMC13077289; doi:10.1111/1751-7915.70349)
Supplement: Supplementary file 1 — Figure S1:. (A). Dependence of magnetite crystal size on the initial iron concentration under standard cultivation conditions. Statistical significance was calculated by a Mann–Whitney test. ns, not significant. (B) Growth of MSR‐1 in presence of different NaNO3 concentrations in 1 mL FSM. Figure S2: Representative profiles of pH (purple triangles) and dissolved oxygen (blue circles) during aerobic growth of 1‐mL culture of MSR‐1 (orange squares) in a microbioreactor system at 28°C with constant vigorous shaking (800 rpm) and an initial pH of 7. Biomass was measured as the intensity of backscattered light (given in arbitrary units [AU]) from a light‐emitting diode. Coloured shading around the symbols indicates the standard deviation calculated from three independent experiments. Figure S3: Representative MSR‐1 cell from the end of the second aerobic cultivation in the seed train. Figure S4: Relationship between magnetosome crystal size and substrate‐uptake rates of MSR‐1 under varying dissolved oxygen (DO) levels. (A) Shows a positive correlation between iron‐uptake rate and magnetite crystal size. (B) No evident correlation between nitrate or lactate uptake rates and magnetite crystal size. Values are given as the mean ± standard deviation calculated from three independent experiments with the same DO level. Figure S5: Correlation between magnetosome crystal size and growth rate of MSR‐1 during cultivation under different DO levels (A) and at different temperatures (B). Solid lines indicate linear regressions of data points with filled symbols (R 2 indicated in the upper left corner) whereas dashed lines represent no change in magnetite crystal size (25.8 nm) as a function of growth rate. Figure S6: Correlation between magnetosome crystal size and abiotic Fe(II) oxidation rates under different DO levels. Oxidation rates were calculated for an initial iron concentration of 30 mM at pH 7.5 according to reference (Kanzaki and Murakami 2013). Table S1: Summary statisti [file MBT2-19-e70349-s001.docx]

**Supplementary Information**

**Controlled low-oxygen supply enables magnetosome size tuning by uncoupling magnetite nucleation and crystal growth in *Magnetospirillum gryphiswaldense***

# **Sophia Tessaro^1^, Markus Schüritz^1,2^, Valérie Jérôme^2^, Ruth Freitag^2^, René Uebe^1,*^**

# ^1^ Department of Microbiology, Faculty of Biology, Chemistry and Geosciences, University of Bayreuth, Universitätsstr. 30, D-95447 Bayreuth, Germany

# ^2^ Department of Process Biotechnology, Faculty of Engineering Sciences, University of Bayreuth, Universitätsstr. 30, D-95447 Bayreuth, Germany

# ^*^ Correspondence: [rene.uebe@uni-bayreuth.de](mailto:rene.uebe@uni-bayreuth.de)

**This Supplementary Information file includes:**

Figures S1 to S6

Tables S1 to S5


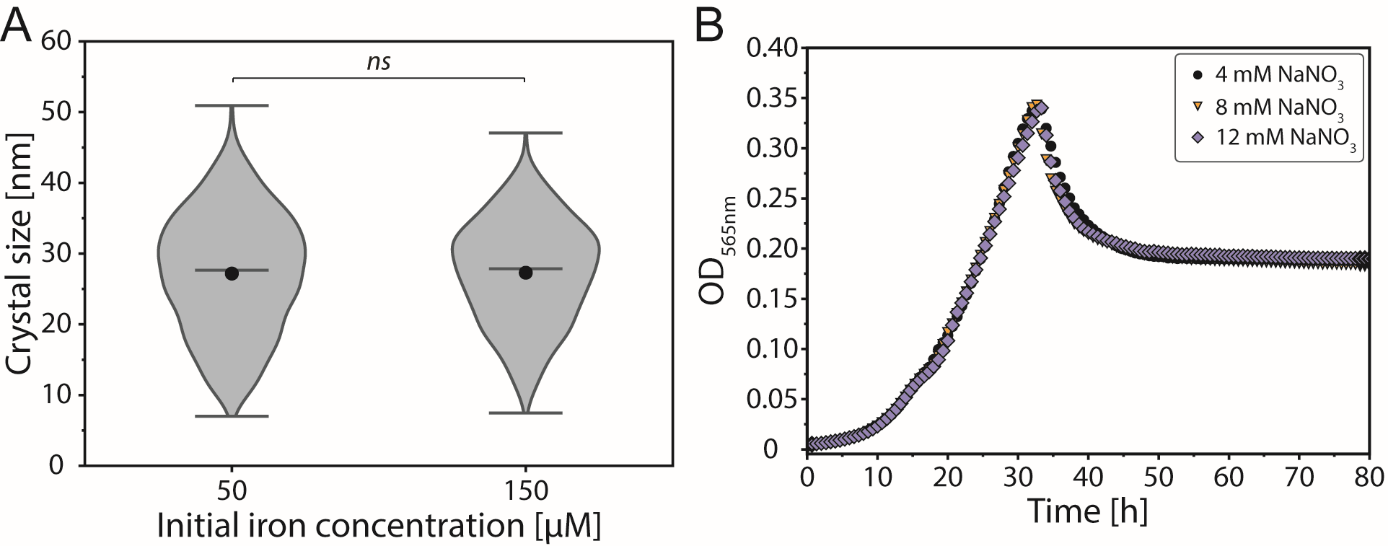


**Fig. S1. (A)** Dependence of magnetite crystal size on the initial iron concentration under standard cultivation conditions. Statistical significance was calculated by a Mann-Whitney test. *ns*, not significant. **(B)** Growth of MSR-1 in presence of different NaNO_3_ concentrations in 1 mL FSM.


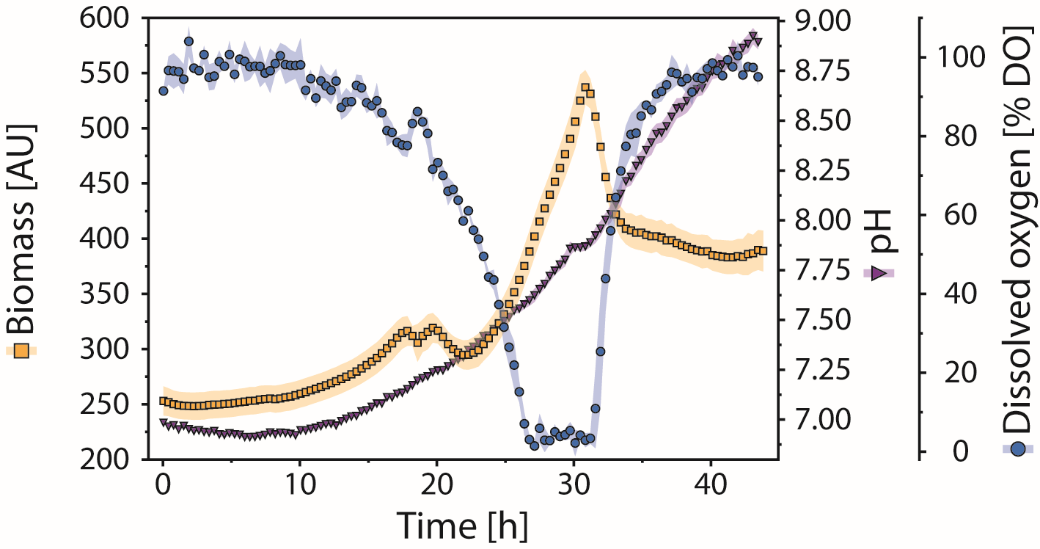


**Fig. S2.** Representative profiles of pH (purple triangles) and dissolved oxygen (blue circles) during aerobic growth of 1-mL culture of MSR-1 (orange squares) in a microbioreactor system at 28 °C with constant vigorous shaking (800 rpm) and an initial pH of 7. Biomass was measured as the intensity of backscattered light (given in arbitrary units (AU)) from a light-emitting diode. Colored shading around the symbols indicates the standard deviation calculated from 3 independent experiments.


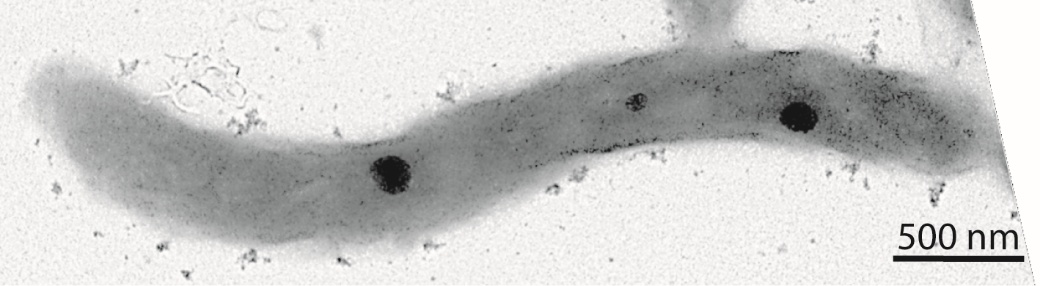


**Fig. S3.** Representative MSR-1 cell from the end of the second aerobic cultivation in the seed train.


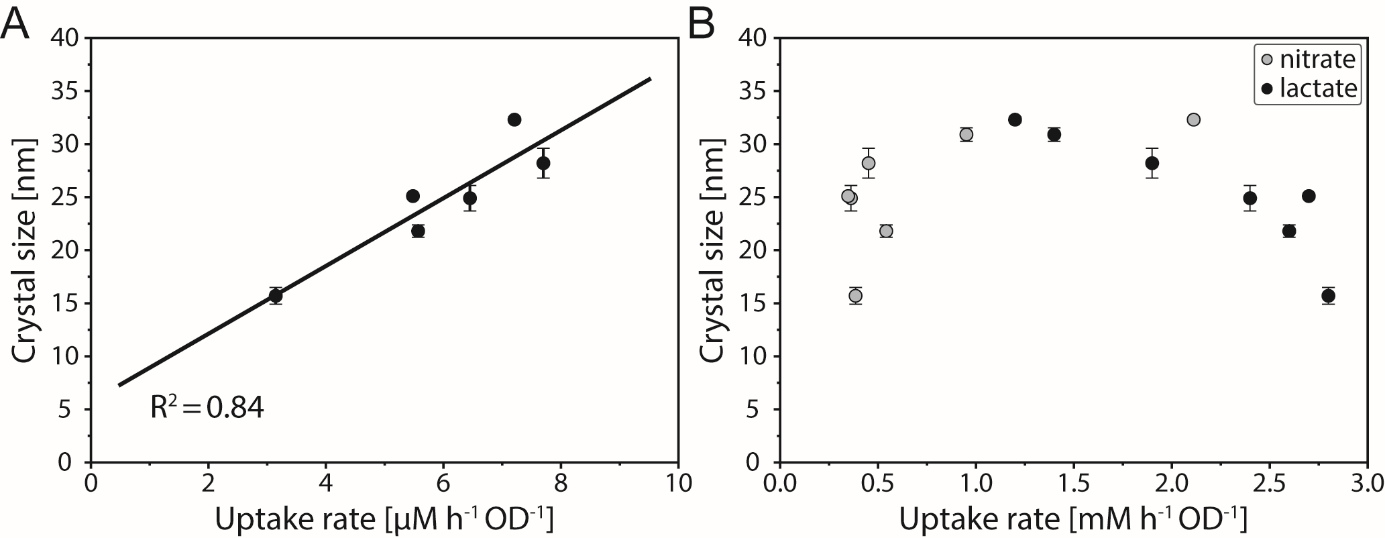


**Fig. S4.** Relationship between magnetosome crystal size and substrate-uptake rates of MSR‑1 under varying dissolved-oxygen (DO) levels. **(A)** Shows a positive correlation between iron-uptake rate and magnetite crystal size. **(B)** No evident correlation between nitrate or lactate uptake rates and magnetite crystal size. Values are given as the mean ± standard deviation calculated from 3 independent experiments with the same DO level.


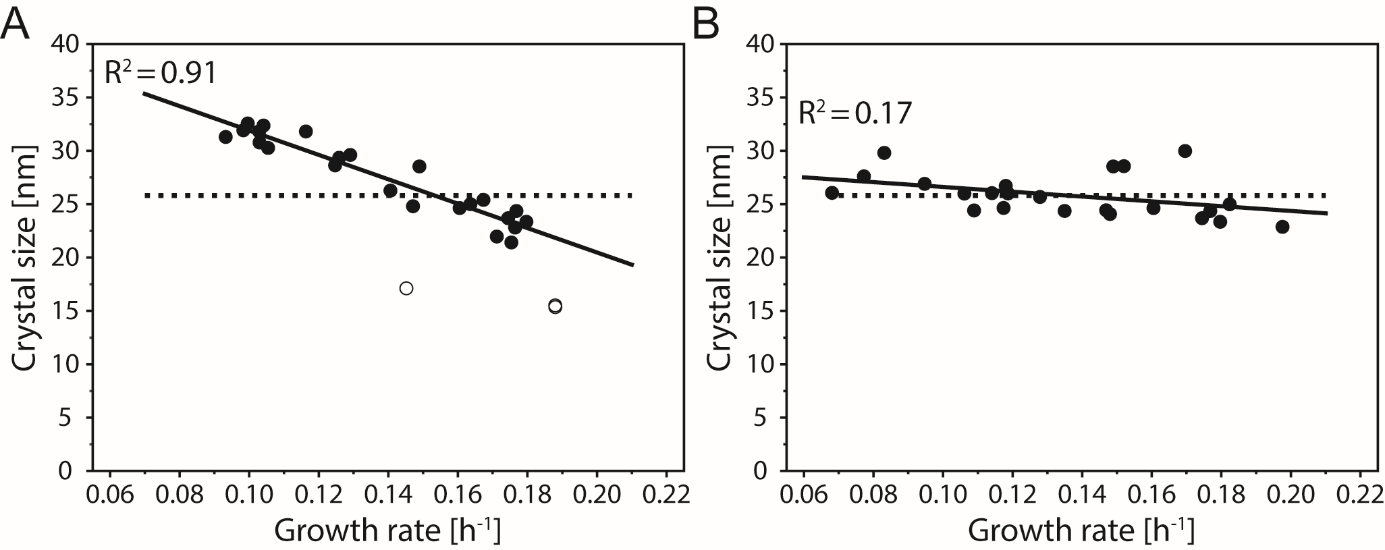


**Fig. S5.** Correlation between magnetosome crystal size and growth rate of MSR-1 during cultivation under different dissolved oxygen (DO) levels **(A)** and at different temperatures **(B)**. Solid lines indicate linear regressions of data points with filled symbols (R^2^ indicated in the upper left corner) whereas dashed lines represent no change in magnetite crystal size (25.8 nm) as a function of growth rate.


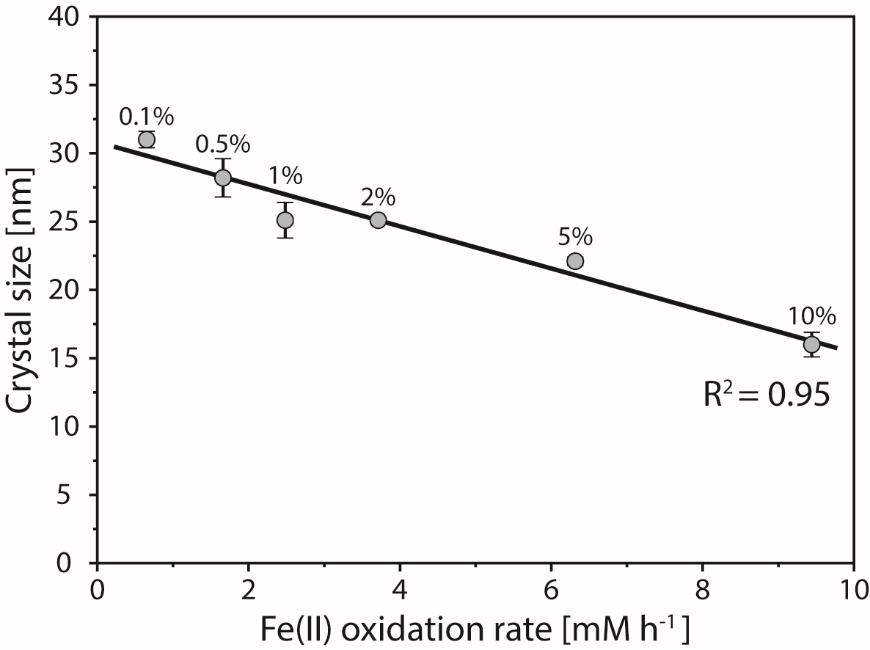


**Fig. S6.** Correlation between magnetosome crystal size and abiotic Fe(II) oxidation rates under different dissolved oxygen (DO) levels. Oxidation rates were calculated for an initial iron concentration of 30 mM at pH 7.5 according to Ref. (Kanzaki and Murakami, 2013).

**Table S1.** Summary statistics for TEM-based magnetosome crystal size quantification for each biological replicate under the DO-dependent cultivation conditions. *, approximate oxygen concentration at the selected DO setpoint.

| DO setpoint [%] | c_O2_ [µM]* | crystal size [nm] | | | | | | sample size | | | |
| --- | --- | --- | --- | --- | --- | --- | --- | --- | --- | --- | --- |
|  |  | mean_pooled_ | replicate 1 | replicate 2 | replicate 3 | mean_replicates_ | SD of means | N_pooled_ | N_replicate 1_ | N_replicate 2_ | N_replicate 3_ |
| 0 | 0 | 32.3 ± 11.1 | 32.4 ± 10.9 | 31.9 ± 10.8 | 32.6 ± 11.6 | 32.3 | 0.3 | 3143 | 1105 | 1025 | 1013 |
| 0.1 | 0.24 | 30.9 ± 10.6 | 30.8 ± 9.8 | 30.3 ± 11.1 | 31.8 ± 10.6 | 31.0 | 0.6 | 3078 | 1018 | 1043 | 1017 |
| 0.5 | 1.22 | 28.2 ± 11.3 | 29.6 ± 10.6 | 28.6 ± 12.3 | 26.3 ± 10.5 | 28.2 | 1.4 | 3087 | 1057 | 1015 | 1015 |
| 1 | 2.44 | 24.9 ± 9.9 | 24.1 ± 8.9 | 24.2 ± 10.8 | 26.9 ± 9.7 | 25.1 | 1.3 | 3681 | 1306 | 1406 | 969 |
| 2 | 4.88 | 25.1 ± 11.2 | 24.8 ± 11.2 | 25 ± 10.4 | 25.4 ± 12 | 25.1 | 0.2 | 2541 | 880 | 820 | 841 |
| 5 | 12.21 | 21.8 ± 7.4 | 22.1 ± 7.5 | 21.4 ± 7.3 | 22 ± 7.3 | 22.1 | 0.3 | 3021 | 1021 | 1018 | 982 |
| 10 | 24.41 | 15.7 ± 6.6 | 17.3 ± 5.4 | 15.4 ± 7.2 | 15.5 ± 6.4 | 16.0 | 0.9 | 1902 | 305 | 617 | 980 |

**Table S2.** Summary statistics for TEM-based magnetosome number per cell quantification for each biological replicate under the DO-dependent cultivation conditions. *, approximate oxygen concentration at the selected DO setpoint.

| DO setpoint [%] | c_O2_ [µM]* | crystal number [cell^-1^] | | | | | | sample size | | | |
| --- | --- | --- | --- | --- | --- | --- | --- | --- | --- | --- | --- |
|  |  | mean_pooled_ | replicate 1 | replicate 2 | replicate 3 | mean_replicates_ | SD of means | n_pooled_ | replicate 1 | replicate 2 | replicate 3 |
| 0 | 0 | 35 ± 9 | 37 ± 8 | 34 ± 9 | 34 ± 9 | 35 | 1 | 90 | 30 | 30 | 30 |
| 0.1 | 0.24 | 39 ± 12 | 42 ± 11 | 36 ± 11 | 41 ± 14 | 40 | 3 | 78 | 24 | 29 | 25 |
| 0.5 | 1.22 | 38 ± 16 | 36 ± 21 | 36 ± 11 | 42 ± 14 | 38 | 3 | 81 | 29 | 28 | 24 |
| 1 | 2.44 | 29 ± 14 | 29 ± 16 | 33 ± 13 | 25 ± 13 | 29 | 3 | 98 | 31 | 32 | 35 |
| 2 | 4.88 | 29 ± 15 | 29 ± 11 | 25 ± 12 | 32 ± 12 | 30 | 4 | 86 | 30 | 32 | 24 |
| 5 | 12.21 | 40 ± 15 | 43 ± 16 | 36 ± 13 | 43 ± 15 | 41 | 3 | 75 | 24 | 28 | 23 |
| 10 | 24.41 | 26 ± 12 | 18 ± 10 | 29 ± 13 | 29 ± 9 | 25 | 5 | 72 | 17 | 21 | 34 |

**Table S3.** Summary statistics for TEM-based magnetosome crystal size quantification for each biological replicate under the temperature-dependent cultivation conditions.

| temperature setpoint [°C] | crystal size [nm] | | | | | | sample size | | | |
| --- | --- | --- | --- | --- | --- | --- | --- | --- | --- | --- |
|  | mean_pooled_ | replicate 1 | replicate 2 | replicate 3 | mean_replicates_ | SD of means | n_pooled_ | replicate 1 | replicate 2 | replicate 3 |
| 18 | 26.9 ± 9.6 | 27.6 ± 9.8 | 27.1 ± 10 | 26.1 ± 9.3 | 26.9 | 0.6 | 1967 | 638 | 714 | 616 |
| 20 | 25.7 ± 9.8 | 26.9 ± 11.6 | 26.0 ± 9.4 | 24.4 ± 9 | 25.8 | 1.0 | 4085 | 790 | 1960 | 1335 |
| 22 | 26.3 ± 8.6 | 26.0 ± 11.4 | 26.0 ± 7.8 | 26.7 ± 8 | 26.3 | 0.3 | 5358 | 956 | 2581 | 1821 |
| 24 | 24.8 ± 9.2 | 25.7 ± 11.3 | 24.6 ± 8 | 24.4 ± 8.3 | 24.9 | 0.6 | 3605 | 1038 | 1193 | 1374 |
| 26 | 26.0 ± 9.4 | 24.9 ± 7.8 | 24.1 ± 7.7 | 28.6 ± 7.7 | 25.9 | 2.0 | 2342 | 715 | 624 | 1003 |
| 28 | 25.1 ± 9.2 | 24.9 ± 9.0 | 25.4 ± 9.8 | 25.2 ± 9.1 | 25.2 | 0.2 | 8350 | 2834 | 1792 | 3724 |
| 30 | 26.0 ± 9.9 | 30 ± 11.5 | 22.9 ± 7.6 | 25.0 ± 9.1 | 25.9 | 3.0 | 3261 | 1063 | 985 | 1213 |

**Table S4.** Summary statistics for TEM-based magnetosome number per cell quantification for each biological replicate under the temperature-dependent cultivation conditions.

| temperature setpoint [°C] | crystal number [cell^-1^] | | | | | | sample size | | | |
| --- | --- | --- | --- | --- | --- | --- | --- | --- | --- | --- |
|  | mean_pooled_ | replicate 1 | replicate 2 | replicate 3 | mean_replicates_ | SD of means | n_pooled_ | replicate 1 | replicate 2 | replicate 3 |
| 18 | 31 ± 12 | 35 ± 13 | 28 ± 12 | 31 ± 12 | 31 | 3 | 97 | 30 | 34 | 33 |
| 20 | 34 ± 12 | 34 ± 12 | 32 ± 7 | 38 ± 17 | 35 | 2 | 119 | 57 | 41 | 21 |
| 22 | 32 ± 12 | 30 ± 11 | 33 ± 11 | 37 ± 13 | 33 | 3 | 167 | 85 | 56 | 26 |
| 24 | 34 ± 13 | 33 ± 15 | 32 ± 11 | 38 ± 10 | 35 | 2 | 103 | 36 | 40 | 27 |
| 26 | 33 ± 16 | 28 ± 11 | 32 ± 8 | 43 ± 16 | 34 | 6 | 100 | 35 | 41 | 24 |
| 28 | 32 ± 12 | 32 ± 11 | 29 ± 13 | 33 ± 13 | 31 | 2 | 262 | 115 | 62 | 85 |
| 30 | 37 ± 16 | 30 ± 11 | 37 ± 11 | 46 ± 21 | 38 | 7 | 88 | 32 | 33 | 23 |

**Table S5.** Summary statistics of process parameters during the active growth phase for each biological replicate (DO- and temperature-setpoint experiments). Values are reported as time-weighted mean ± time-weighted mean absolute deviation.

| Cultivation | | DO [%] | | | | Temp [°C] | | | | pH | | | |
| --- | --- | --- | --- | --- | --- | --- | --- | --- | --- | --- | --- | --- | --- |
|  |  | mean | replicate 1 | replicate 2 | replicate 3 | mean | replicate 1 | replicate 2 | replicate 3 | mean | replicate 1 | replicate 2 | replicate 3 |
| Dissolved Oxygen [%] | 0 | 0.00 ± 0.00 | 0.00 ± 0.00 | 0.00 ± 0.00 | 0.00 ± 0.00 | 28 ± 0.006 | 28 ± 0.005 | 28 ± 0.005 | 28 ± 0.008 | 7.00 ± 0.06 | 6.99 ± 0.06 | 7.00 ± 0.06 | 7.01 ± 0.05 |
|  | 0.1 | 0.10 ± 0.03 | 0.09 ± 0.03 | 0.10 ± 0.02 | 0.10 ± 0.03 | 28 ± 0.003 | 28 ± 0.004 | 28 ± 0.003 | 28 ± 0.005 | 7.02 ± 0.04 | 7.01 ± 0.04 | 7.03 ± 0.03 | 7.02 ± 0.03 |
|  | 0.5 | 0.45 ± 0.10 | 0.45 ± 0.10 | 0.45 ± 0.08 | 0.44 ± 0.12 | 28 ± 0.011 | 28 ± 0.019 | 28 ± 0.006 | 28 ± 0.007 | 7.02 ± 0.03 | 7.03 ± 0.03 | 7.02 ± 0.03 | 7.03 ± 0.03 |
|  | 1 | 0.93 ± 0.13 | 0.93 ± 0.15 | 0.93 ± 0.12 | 0.93 ± 0.11 | 28 ± 0.016 | 28 ± 0.013 | 28 ± 0.019 | 28 ± 0.015 | 7.03 ± 0.03 | 7.03 ± 0.04 | 7.03 ± 0.03 | 7.03 ± 0.03 |
|  | 2 | 1.92 ± 0.20 | 1.92 ± 0.19 | 1.92 ± 0.26 | 1.93 ± 0.15 | 28 ± 0.006 | 28 ± 0.005 | 28 ± 0.005 | 28 ± 0.008 | 7.02 ± 0.04 | 7.02 ± 0.03 | 7.03 ± 0.03 | 7.01 ± 0.05 |
|  | 5 | 4.90 ± 0.24 | 4.91 ± 0.22 | 4.90 ± 0.27 | 4.89 ± 0.23 | 28 ± 0.006 | 28 ± 0.006 | 28 ± 0.006 | 28 ± 0.006 | 7.03 ± 0.03 | 7.04 ± 0.04 | 7.03 ± 0.03 | 7.03 ± 0.03 |
|  | 10 | 9.91 ± 0.28 | 9.93 ± 0.26 | 9.90 ± 0.27 | 9.90 ± 0.31 | 28 ± 0.006 | 28 ± 0.006 | 28 ± 0.006 | 28 ± 0.007 | 7.04 ± 0.04 | 7.03 ± 0.03 | 7.04 ± 0.04 | 7.04 ± 0.04 |
| Temperature [°C] | 18 | 1.05 ± 0.24 | 1.01 ± 0.14 | 1.15 ± 0.43 | 0.99 ± 0.14 | 18 ± 0.008 | 18 ± 0.007 | 18 ± 0.007 | 18 ± 0.008 | 7.02 ± 0.06 | 7.00 ± 0.03 | 7.03 ± 0.12 | 7.03 ± 0.04 |
|  | 20 | 0.99 ± 0.11 | 0.99 ± 0.11 | 0.98 ± 0.09 | 1.00 ± 0.14 | 20 ± 0.005 | 20 ± 0.009 | 20 ± 0.004 | 20 ± 0.004 | 7.03 ± 0.04 | 7.02 ± 0.04 | 7.04 ± 0.04 | 7.04 ± 0.04 |
|  | 22 | 0.97 ± 0.12 | 0.98 ± 0.16 | 0.97 ± 0.11 | 0.96 ± 0.10 | 22 ± 0.005 | 22 ± 0.007 | 22 ± 0.003 | 22 ± 0.003 | 7.04 ± 0.04 | 7.05 ± 0.05 | 7.04 ± 0.04 | 7.04 ± 0.04 |
|  | 24 | 0.95 ± 0.12 | 0.97 ± 0.10 | 0.93 ± 0.13 | 0.96 ± 0.12 | 24 ± 0.004 | 24 ± 0.007 | 24 ± 0.003 | 24 ± 0.003 | 7.01 ± 0.05 | 7.05 ± 0.05 | 7.03 ± 0.03 | 6.95 ± 0.05 |
|  | 26 | 0.98 ± 0.18 | 0.95 ± 0.11 | 0.92 ± 0.17 | 1.07 ± 0.27 | 26 ± 0.005 | 26 ± 0.008 | 26 ± 0.004 | 26 ± 0.004 | 7.03 ± 0.03 | 7.02 ± 0.03 | 7.04 ± 0.04 | 7.04 ± 0.04 |
|  | 28 | 0.93 ± 0.15 | 0.93 ± 0.15 | 0.92 ± 0.18 | 0.95 ± 0.13 | 28 ± 0.014 | 28 ± 0.015 | 28 ± 0.013 | 28 ± 0.015 | 7.03 ± 0.03 | 7.03 ± 0.03 | 7.03 ± 0.04 | 7.03 ± 0.03 |
|  | 30 | 1.04 ± 0.29 | 0.93 ± 0.18 | 1.28 ± 0.32 | 0.91 ± 0.36 | 30 ± 0.010 | 30 ± 0.019 | 30 ± 0.005 | 30 ± 0.005 | 7.04 ± 0.04 | 7.04 ± 0.04 | 7.04 ± 0.04 | 7.03 ± 0.03 |
